# Effects of plant-based proteins and handling stress on intestinal mucus microbiota in rainbow trout

**DOI:** 10.1038/s41598-023-50071-x

**Published:** 2023-12-19

**Authors:** Marvin Suhr, Finn-Thorbjörn Fichtner-Grabowski, Henrike Seibel, Corinna Bang, Andre Franke, Carsten Schulz, Stéphanie C. Hornburg

**Affiliations:** 1 Institute of Animal Nutrition and Physiology, Christian-Albrechts-University Kiel, Hermann-Rodewald-Straße 9, 24118 Kiel, Germany; 2 Fraunhofer Research Institution for Individualized and Cell-Based Medical Engineering (IMTE), Hafentörn 3, 25761 Büsum, Germany; 3 Institute of Clinical Molecular Biology, Christian-Albrechts-University Kiel, University Hospital Schleswig-Holstein, Rosalind-Franklin-Str. 12, 24105 Kiel, Germany; 4 Institute of Animal Breeding and Husbandry, Christian-Albrechts-University Kiel, Hermann-Rodewald-Straße 6, 24118 Kiel, Germany

**Keywords:** Microbiome, Animal physiology

## Abstract

Via 16S rRNA gene amplicon sequencing, this study explores whether the gut mucus microbiota of rainbow trout is affected by the interaction of a plant-protein-based diet and a daily handling stressor (chasing with a fishing net) across two genetic lines (A, B). Initial body weights of fish from lines A and B were 124.7 g and 147.2 g, respectively. Fish were fed 1.5% of body weight per day for 59 days either of two experimental diets, differing in their fish meal [fishmeal-based diet (F): 35%, plant-based diet (V): 7%] and plant-based protein content (diet F: 47%, diet V: 73%). No diet- or stress-related effect on fish performance was observed at the end of the trial. However, we found significantly increased observed ASVs in the intestinal mucus of fish fed diet F compared to diet V. No significant differences in Shannon diversity could be observed between treatments. The autochthonous microbiota in fish fed with diet V was dominated by representatives of the genera *Mycoplasma*, *Cetobacterium,* and *Ruminococcaceae,* whereas *Enterobacteriaceae* and *Photobacterium* were significantly associated with diet F. The mucus bacteria in both genetic lines were significantly separated by diet, but neither by stress nor an interaction, as obtained via PERMANOVA. However, pairwise comparisons revealed that the diet effect was only significant in stressed fish. Therefore, our findings indicate that the mucus-associated microbiota is primarily modulated by the protein source, but this modulation is mediated by the stress status of the fish.

## Introduction

The aquaculture industry has become the world’s largest contributor of nutritious aquatic food for human consumption^1^. With global efforts to reduce the use of fish meal (FM) and fish oil (FO) from wild capture fisheries in aquafeeds^2^, there has been a profound development in the last two decades in the partial substitution of conventional FM with plant-based ingredients, insect meal, or animal by-products^3–5^. In carnivorous fish, such as rainbow trout, 20–40% of the FM fraction can be replaced by soybean meal (SBM), for example, with little effect on growth, gut health, and nutrient utilization^6–8^. While soybean products have a relatively high crude protein (CP) content and a favorable amino acid profile, high amounts of carbohydrates and antinutritive factors (ANF) have previously been associated with adverse effects such as low digestibility, induction of mild to severe enteritis^6^, and malfunction of the intestinal mucosa in carnivorous fish^9^. However, the use of highly processed soybean products, such as soybean protein concentrates (SPC), reduces these adverse effects, thereby increasing their use in aquafeeds^10–12^.

In intensive aquaculture, potential (handling) stress, such as stocking density, netting, grading, transportation, and handling^13^, are ubiquitous, and several physiological biomarkers such as growth, behavior, histological, and immunological parameters for early indication of stress in fish exist^14–16^. A consequence of such stimuli may lead to a disbalance in homeostasis or allostasis, which is followed by a negative physiological and behavioral response (stress), and can lead to the loss of appetite, overall performance, immunosuppression, reduced reproductivity, and cognitive ability^17^. Especially in mammals, it is well established that stress is able to disrupt the microbial (gut) communities via several vectors, such as hormones and immune factors secreted by the host or the bi-directional communication of the nervous system and peripheral intestinal functions known as the gut-brain axis^18^. It is likely that similar interactions dictate the condition in other vertebrates, including fish.

It is now generally accepted that environmental factors and feeding strategies mediate the profile of the microbiome, and studies on several salmonid species and alternative diets exist^6,19–24^. However, there is still a substantial lack of information about the influence of stress on the microbiome and its interaction with the fish host. In a 7-month feeding trial, rainbow trout were fed a plant-based diet with a main substitution of FM and FO with plant proteins and oils, high cortisol values, during which apathetic and stress-related behavior was observed^25^. Contrary to this, another study revealed no measurable stress response based on cortisol, glucose, and lysozyme plasma levels when rainbow trout were fed a FM and crude protein-reduced diet combined with applied handling stress^26^. We previously observed that the expression of molecular stress response markers such as *tumor necrosis factor* (*TNF*)*α* and immunoglobulins^27^ are downregulated in rainbow trout fed with a plant-based diet while exposed to an external stressor compared to trout fed with a FM-based diet. These results demonstrate the potential impact of plant-based diets in combination with handling stress on fish health, physiology, and welfare.

From a holistic perspective, the intestinal microbiome can represent a natural mediator in this system. Especially the mucus layer, whose interface is proposed to be of major importance for host health due to the interactions with adherent bacteria. Those autochthonous bacteria are embedded within a viscoelastic matrix containing water, gel-forming mucin glycoproteins, lipids, and several proteins^28^. Such bacteria are attributed with assisting functions such as gut health, nutrient metabolism, pathogen protection, and host immunity^29^. The evolution of an adaptive immune system along mucosal tissues has been proposed to be the conclusion of a long-term interlaced interaction of symbiotic microorganisms and host factors^30,31^. It is now generally acknowledged that the gut microbiome represents an important driver of animal welfare and fish performance^32^. Thus, a balanced and functional mucosal epithelium populated with commensal microbes is vital for fish health, and an imbalance can cause dysbiosis and malfunction in this multi-individual system. However, studies related to stress as a mediator on the intestinal mucus microbiota of rainbow trout are scarce to date. Therefore, understanding the microbiome of the alimentary tract with all its sub-microcompartments under different feeding and stress conditions has to be considered for analysis by using high-throughput sequencing techniques.

Consequently, the present study (i) aimed to characterize the resident microbiota of the intestinal mucosa of rainbow trout from two different trout genetic lines via 16S rRNA gene sequencing fed with either a FM-based diet or a plant-based diet. We hypothesized that the diet type interacts with an external stressor when modulating the mucus microbiota of trout. Therefore, we (ii) aimed to further induce chronic stress in rainbow trout by chasing the fish twice daily with a fishing net inside the tank and evaluated the interaction of diet type and stress on the mucosal bacteria. In a recently published study^33^, we investigated the allochthonous microbiota in the gut contents obtained from the same individual trout, where an interaction of stress and diet led to a significant change in the composition and abundance of its related bacterial communities. Based on these results, we (iii) decided to evaluate the effects of diet and stress on the different intestinal compartments in a meta-study combining both datasets.

## Results

### Growth performance of rainbow trout

We used two different genetic lines (A and B) of female rainbow trout in a 59-day experiment to assess the influence of a plant-based diet (V) in comparison to a rather fishmeal-based diet (F) in combination with simulated handling stress on the mucosal gut microbiota. We additionally assessed classical performance parameters, including specific growth rate (SGR), feed conversion ratio (FCR), daily feed intake (DFI), protein efficiency ratio (PER), and protein retention efficiency (PRE), based on group measures from feeding-day 50. Neither the substitution of FM with plant-based ingredients nor the simulated handling stress significantly affected fish growth performance or nutrient utilization (Supplementary File S2 Table 1), apart from daily feed intake, which was significantly lower (*p* < 0.001) in fish fed the fishmeal diet F (1.54 ± 0.02) than in fish fed the plant-protein-based diet V (1.50 ± 0.01). However, a difference in daily feed intake of 0.04% is not considered biologically relevant. The average SGR was 1.75 ± 0.05 for trout genetic line A and 1.76 ± 0.05 for trout genetic line B. A detailed discussion of fish growth results from the same experiment is published and discussed elsewhere^27,33^.

### 16S rRNA gene sequencing analysis

A total of 209 samples, collected from the intestinal mucosa of two breeding lines of rainbow trout and 10 extraction controls, were sequenced using the V3-V4 16S rRNA primer on an Illumina MiSeq platform, which generated 12.2 million reads in both directions with an amplicon length of 2 × 300 bp. After ASV clustering, mitochondrial and chloroplast reads were removed from the dataset and subsequently quality-filtered resulting in 178 samples that were further analyzed. 72 (A) and 75 (B) samples from the end of the trial and 19 (A-init) and 12 (B-init) initial samples (Supplementary File S2 Table 2) were sustained in the data set. Of the total 5,813,585 raw reads, excluding the technical controls, 3,659,931 filtered reads, with 168 unique ASVs, were left for downstream analysis.

Positive controls (mock samples) were included in the sequencing run and evaluated for accurate taxonomical identification at the species level and quantitative measures of defined bacteria (Supplementary File S1, Fig. S1).

### Dietary effect on alpha diversity

**Figure 1:**
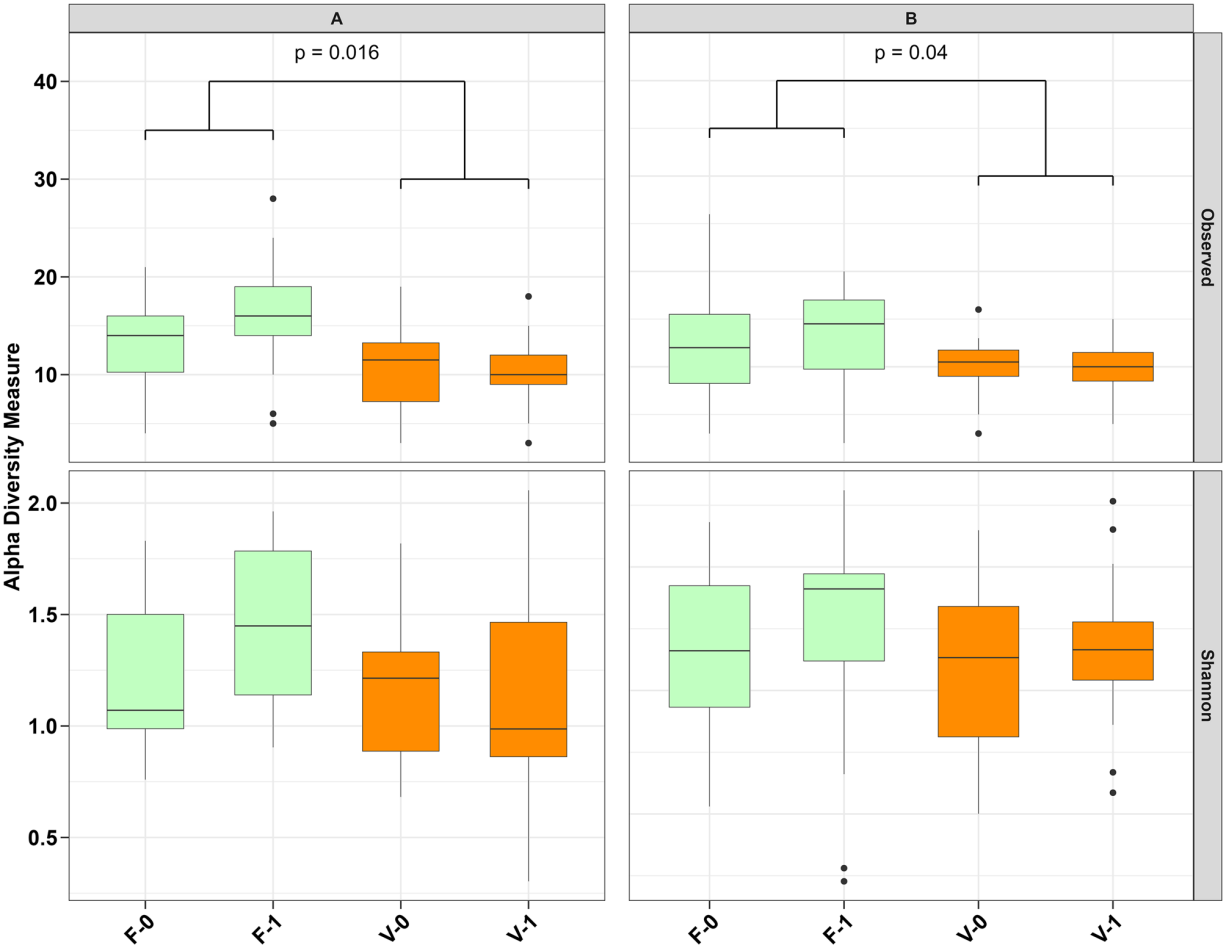
Alpha diversity indices in relation to the treatment group. Alpha diversity indices, including Observed ASVs (upper panels) and Shannon diversity index (lower panels) for intestinal mucosal tissue of trout genetic lines A (left panels) and B (right panels). Boxes are colored according to the experimental diet (F: fish meal diet, light green; V: plant-based diet, orange). Stressed group (1) and unstressed group (0). n = 3.

Evaluation of alpha diversity parameters revealed an overall low richness and Shannon diversity in the intestinal mucosa in both trout genetic lines and all treatments. Figure 1 shows the mean values of alpha diversity measures (Observed and Shannon) among dispersion parameters. The alpha diversity parameters of each trout line were tested with a two-way ANOVA using a linear mixed-effect model with diet and stress as main factors and both as interaction factors. Tanks were considered random factors. In both trout genetic lines, we observed a significantly increased richness in fish fed diet F in comparison to diet V (trout line A: p = 0.016; trout line B: p = 0.04, Supplementary File S2 Table 3). However, taking into account species evenness, no significant differences between treatments in Shannon diversity indices could be observed. Although trout lines were not statistically compared, both have alpha diversity within the same magnitude, suggesting similar bacterial richness and evenness.

### Effects of diet and stress on beta diversity of the gut mucus community

**Figure 2:**
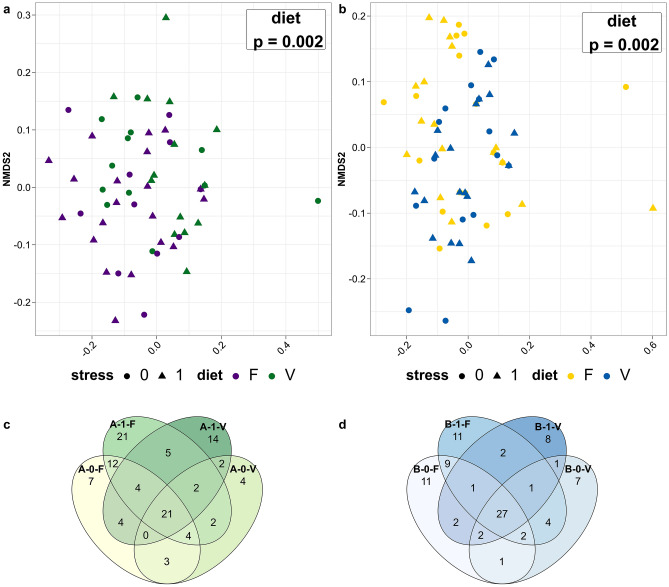
Beta diversity metrics indicating diet as a factor for microbial clustering. (**a**) and (**b**) Non-metric multi-dimensional scaling (NMDS) plot of unweighted UniFrac distances that incorporate phylogenetic information and presence/absence data from the mucus of the intestinal gut of rainbow trout lines A (**a**) and B (**b**) based on genus-level data. Dots are colored according to the factor diet and shaped according to the factor stress. Bacterial communities in both breeding lines cluster significantly by the factor diet indicated by p-value in the top right corner of the graph. (**c**) and (**d**) Number of shared ASVs between treatment groups in trout genetic line A (**c**, green) and B (**d**, blue). Unstressed group (0), stressed group (1), fishmeal diet (F), plant-based diet (V).

To explore differences in bacterial communities obtained from rainbow trout gut mucus explained by diet or stress, phylogenetically unweighted and weighted UniFrac distances were calculated based on genus level and represented by non-metric multi-dimensional scaling (NMDS) for unweighted distances (Fig. 2). By using the adonis function, a permutational analysis of variance (PERMANOVA, Table 1) was performed to test for the main effects of diet and stress, and interactive effects in the respective trout genetic line. Unweighted and weighted UniFrac distances in trout line A revealed that the microbial communities are significantly separated when considering diet as a factor (PERMANOVA, unweighted: p = 0.002, weighted: p = 0.001) and no effects were detected for stress as well as no interaction effects. This diet effect is significantly pronounced in fish that were stressed (pairwise comparison MANOVA, Table 2: unweighted: p = 0.002, weighted: p = 0.05). Congruently, testing for differences in trout line B for the main effects and interactions revealed diet as the main factor modulating the microbial communities in both distances (PERMANOVA unweighted: p = 0.002, weighted: p = 0.021). It was also confirmed by pairwise comparison that the diet effect occurred only in stressed fish (Pairwise comparison MANOVA, Table 2: unweighted: p = 0.034). This is in accordance with the clustering of communities by the factor diet (visualized by colors) in Fig. 2a and b and the results from the analysis of similarities (ANOSIM, Table 1). A multivariate test for homogeneity in (beta) dispersion applied for diet and stress on both distances of each trout line revealed equal dispersion except for factor diet in weighted distances of trout line A (p = 0.045) (Supplementary File S2 Table 4).

**Table 1:** Results of PERMANOVA analysis using adonis function and anosim on unweighted and weighted UniFrac distances of intestinal gut mucus microbiota of two rainbow trout genetic lines (A and B) with factors diet (fishmeal diet F, plant-based diet V) and stress (unstressed group 0, stressed group 1).

	Unweighted				Weighted			
	Adonis		ANOSIM		Adonis		ANOSIM	
	p-value	R^2^	p-value	R^2^	p-value	R^2^	p-value	R^2^
A								
Stress	0.755	0.007	0.856	− 0.021	0.935	0.004	0.434	− 0.001
Diet	**0.002**	0.067	**0.002**	0.095	**0.001**	0.081	**0.003**	0.117
Stress * diet	0.082	0.025	–	–	0.826	0.006	–	–
B								
Stress	0.967	0.003	0.818	− 0.016	0.706	0.006	0.693	− 0.011
Diet	**0.002**	0.056	**0.001**	0.098	**0.021**	0.053	**0.006**	0.085
Stress * diet	0.865	0.006	–		0.933	0.003	–	–

Significant values are in bold.

**Table 2:** Pairwise comparison using permutation MANOVA of unweighted and weighted UniFrac distances of intestinal gut mucus microbiota of two rainbow trout genetic lines (A and B), using treatment as a factor.

	Unweighted	Weighted
	p-value	p-value
A		
0-V:0-F	0.485	**0.050**
1-V:1-F	**0.002**	**0.050**
0-V:1-V	0.510	1
0-F:1-F	0.354	1
B		
0-V:0-F	0.071	0.182
1-V:1-F	**0.034**	0.182
0-V:1-V	1	1
0-F:1-F	1	1

P-values were manually adjusted with method holm. Unstressed group (0), stressed group (1), fishmeal diet (F), plant-based diet (V).

Significant values are in bold.

### Structure of the autochthonous community of bacteria in the gut intestinal

**Figure 3:**
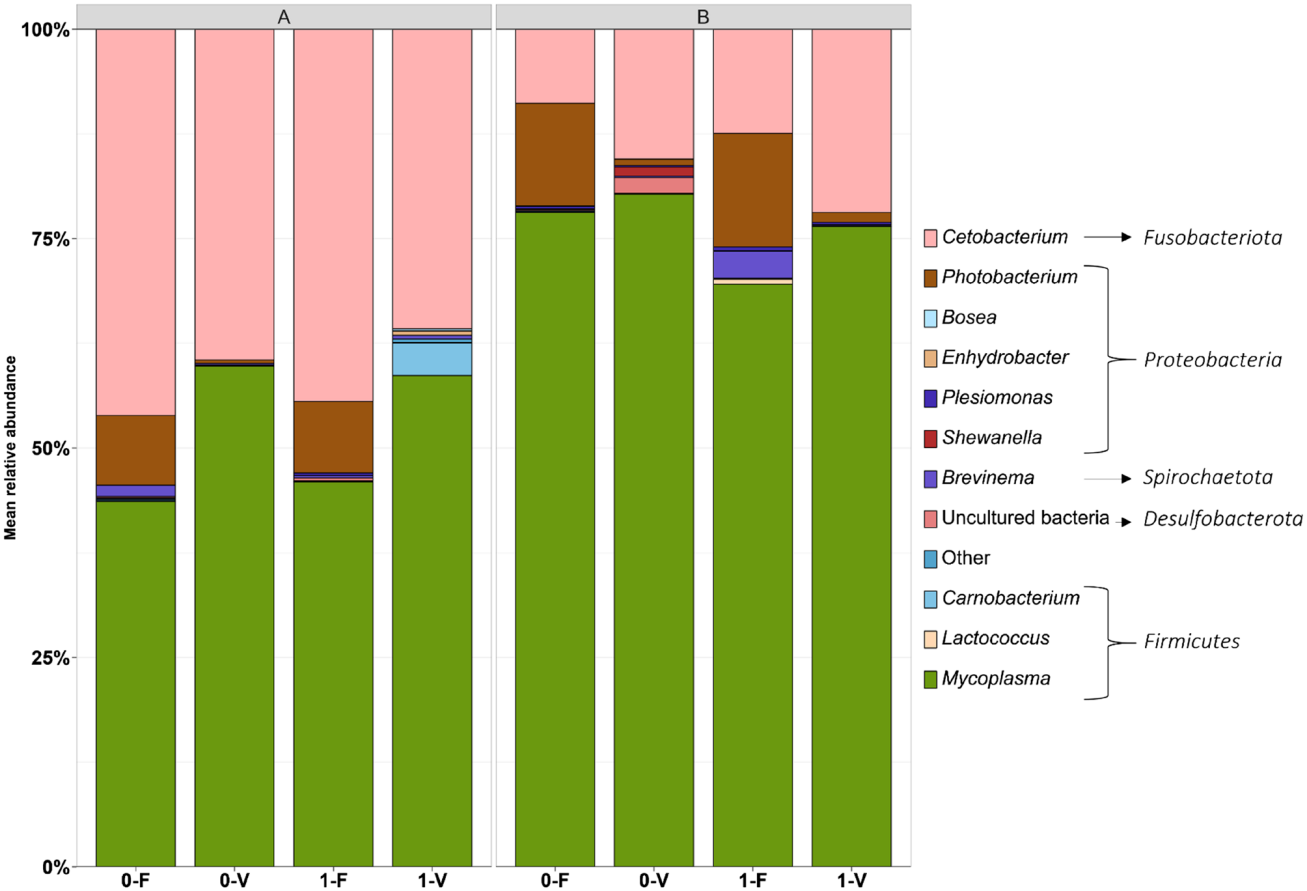
Microbial composition of the intestinal gut mucosa. The four respective bars represent a diet-stress combination after the 59-day trial from both trout genetic lines, and the frequency of bacteria is indicated as the mean relative abundance. The data is based on the taxonomic rank genus. Category ‘Other’ implies taxonomical clades with an overall abundance of < 0.15%. The order of the bars is arranged by abundance, except for the most abundant genus, which is placed at the bottom for legibility. Each treatment incorporates individual fish data from the three (N = 3) tanks. Unstressed group (0), stressed group (1), fishmeal diet (F), plant-based diet (V).

The relative abundance of bacteria isolated from the intestinal gut mucosa of rainbow trout revealed a community dominated by the phyla *Fusobacteriota*, *Proteobacteria*, *Actinobacteriota*, *Desulfobacterota*, *Spirochaetota,* and *Firmicutes* (Fig. 3, Supplementary File S2 Fig. S2, Supplementary File S2 Table 5)*.* The category “Other” implies entities that are represented by less than 0.05%. On the basis of mean relative abundance, *Firmicutes* and *Fusobacteriota* represent the most abundant phyla across all treatments, including trout lines A and B.

On the genus level, the microbial communities are overrepresented by *Mycoplasma* from the phylum *Firmicutes* in trout lines A (mean: 52.0%) and trout line B (mean: 76.1%). In trout line A, higher abundance was observed in diet V independent of stress (A-0-V mean: 59.8%; A-1-V mean: 58.6%) compared to diet F in both stress groups (A-0-F mean: 43.6%; A-1-F mean: 45.9%). This was confirmed to be significant by linear discriminant analysis of effect size (LEfSe, diet: p = 0.001, Table 3). In trout line B, *Mycoplasma* abundance was enriched in fish that were not stressed (mean: 65.5%) compared to fish that were stressed (mean: 62.6%). Moreover, a significantly higher abundance (LEfSe, diet: p = 0.011, Table 3) in fish fed diet V was observed. The second most abundant genus from the phylum *Firmicutes* was *Carnobacterium,* which was only present in trout line A and not in trout line B. Here, stressed fish fed with diet V had the highest abundance (mean: 3.8%). Considering relative abundance, *Cetobacterium*, the single representative of the phylum *Fusobacteriota,* was found to be the second most abundant genus in trout lines A (mean: 41.4%) and B (mean: 14.7%). In trout line A, *Cetobacterium* was more abundant in fish fed diet F (not significant) compared to diet V. An obverse effect is observed in trout line B, where *Cetobacterium* abundance is increased when fed with diet V, which was confirmed by LEfSe analysis (p < 0.001). In both trout lines, *Photobacterium,* from the phylum *Proteobacteria,* is significantly promoted when provided with the FM-based diet compared to the plant-based diet (LEfSe, A, diet: p < 0.001; B, diet: p < 0.001; Table 3).

**Table 3:** Linear discriminant analysis effect size (LEfSe) results that explain significantly different (p < 0.05) genera in the gut mucus microbiota of rainbow trout between diet types.

Trout genetic line	Genus	enrich_group	ef_lda	pvalue	padj
A	Photobacterium	F	5.0666	< 0.001	< 0.001
	Plesiomonas	F	4.4470	< 0.001	< 0.001
	Desulfovibrionaceae_uncultured	F	4.0606	0.0405	0.0405
	Lactobacillus	F	3.9885	0.0073	0.0073
	Crenobacter	F	3.8322	0.0253	0.0253
	Enterobacteriaceae	F	3.7741	0.0470	0.0470
	Mycoplasma	V	5.0496	0.0012	0.0012
B	Photobacterium	F	5.1655	< 0.001	< 0.001
	Desulfovibrionaceae	F	4.6168	0.0021	0.0021
	Lactococcus	F	4.4111	0.0005	0.0005
	Enterobacteriaceae	F	4.3175	0.0018	0.0018
	Cetobacterium	V	5.0332	< 0.001	< 0.001
	Mycoplasma	V	4.8299	0.0110	0.0110
	Ruminococcaceae	V	4.4533	0.0385	0.0385

Furthermore, in trout line A, *Plesiomonas*, *Lactobacillus*, *Crenobacter,* and two unclassified genera from the family *Desulfovibrionaceae* and *Enterobacteriaceae,* and in trout line B, *Lactococcus* and three unclassified genera from the family *Desulfovibrionaceae* and *Enterobacteriaceae* were significantly enriched in diet F (Table 3). An unclassified genus from the *Ruminococcaceae* was found to be enriched in diet V in trout line B. In addition, we identified *Lactococcus, Plesiomonas, Bosea, Brevinema, Shewanella,* and *Enhydrobacter* as representatives with an overall low abundance (< 0.05%)*. Shewanella* was identified as a unique genus in the unstressed group fed with diet V in trout line B. The remaining genera displayed are of less than 4% mean abundance for each treatment and are not found to be different among the treatments. A total of 21 ASVs in trout line A (Fig. 2c) are shared among all four treatments, with *Mycoplasma*, *Cetobacterium*, *Brevinema*, *Enhydrobacter,* and *Cutibacterium* as the dominant representative genera. In trout line B, 27 ASVs intersect between treatments; were in addition to the genera in trout line A, *Photobacterium, Plesimonas*, *Mesorhizobium,* and *Harryflintia* are shared (Fig. 2d).

### Microbiota comparison between samples from gut mucus and gut contents

**Figure 4:**
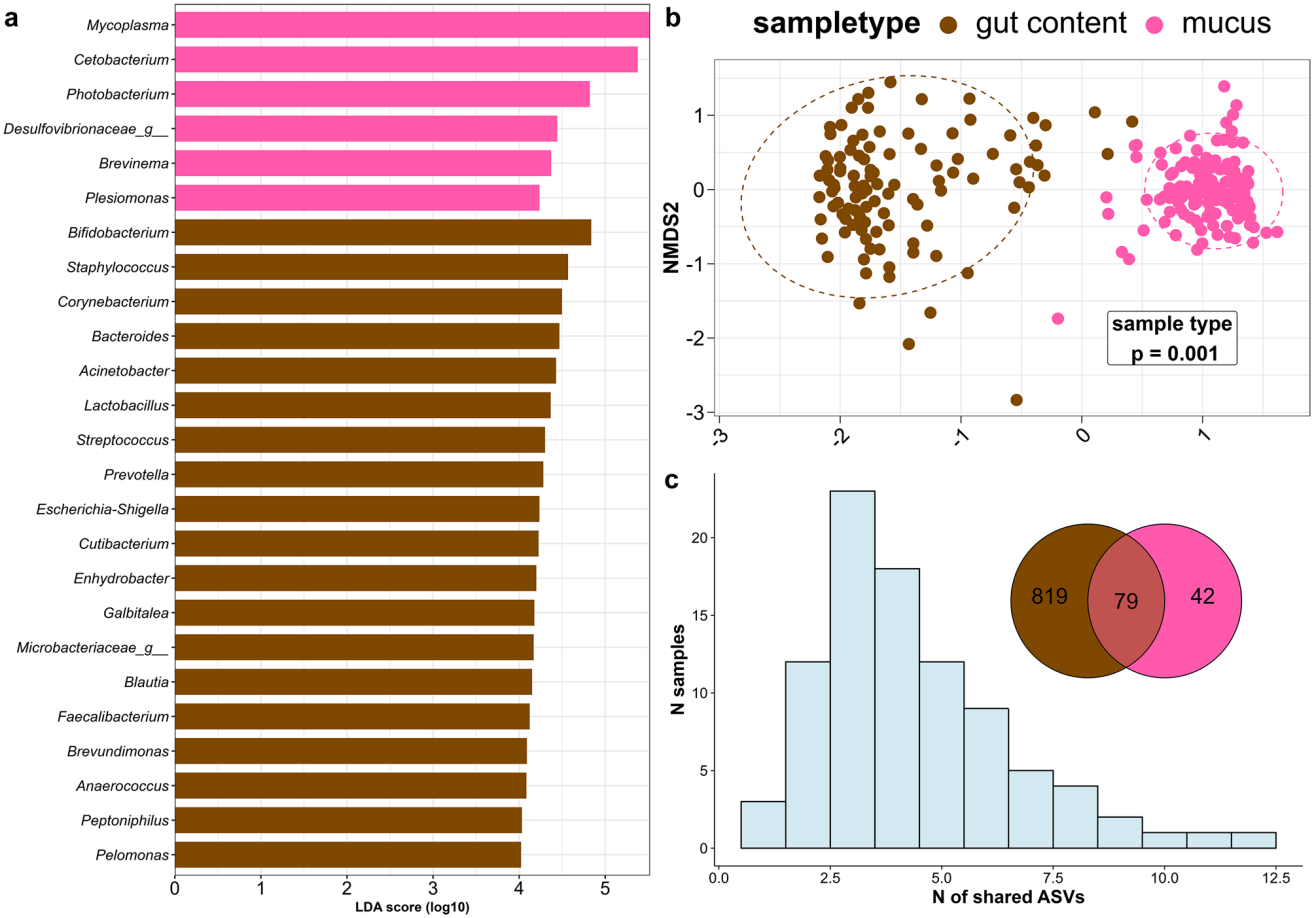
Comparing the microbiota of intestinal sub-compartments mucus (pink) and gut contents (brown). Samples were analyzed according to the sample type, regardless of diet, stress, or trout line. LEfSe was conducted with sample type as the group factor (**a**). NMDS of Bray–Curtis distance reveals significant differences in bacterial community composition (**b**). (**c**) Shows a histogram where the number of shared ASVs against the number of samples is presented and a VENN diagram with observed ASVs in sample types gut content, mucus, and shared fraction. Data from gut content samples are obtained from^33^.

We used previously published^33^ 16S rRNA amplicon sequence data derived from the gut contents of the same individual trout from this experimental trial to compare the microbial structure of the gut mucosa in a meta-analytical approach. Information about diet, stress, or trout genetic line was neglected to focus on differences between the sampling sites of the intestine of rainbow trout. Individual communities from gut contents (N = 104) and mucus (N = 147) were examined using NMDS on Bray–Curtis distances on genus-level data (Fig. 4b). Samples clustered according to their sample type, which was confirmed by PERMANOVA using sampletype as a factor (p = 0.001), suggesting significant differences in their microbial composition despite the effects of diet, stress, and trout line observed in previous results. Differences between the sample types were also observed in alpha diversity, where significantly increased values of observed ASVs (Wilcoxon, p = 1e−38) and Shannon diversity indices (Wilcoxon, p = 2.3e−21, Supplementary File S1 Fig. S5) were accounted for samples extracted from gut contents. Figure 4c shows a Venn diagram where 819 and 42 ASVs were detected in contents and mucus, respectively. In total, 79 ASVs overlap in both sample type communities, with the maximum number of 3 shared ASVs (Fig. 4c, histogram: N of shared ASVs) being observed in 23 samples (N samples). To identify which bacteria contributed the most to the respective group variable sample type, a LEfSe analysis was performed on this dataset (Fig. 4a). The genera *Mycoplasma*, *Cetobacterium*, *Photobacterium, Brevinema, Plesiomonas,* and unclassified bacteria from *Desulfovibrionaceae* (LDA score > 4.0) accounted for greater relative abundance in samples from mucus compared to gut contents, where *Bifidobacterium*, *Staphylococcus*, *Corynebacterium*, *Bacteroides*, *Acinetobacter,* and *Lactobacillis* (LDA score > 4.0) were more abundant.

## Discussion

Alternative protein sources that replace fishmeal remain a controversial and important research subject in aquaculture, fish nutrition, and animal husbandry. On the other hand, sustaining husbandry conditions in aquaculture, which entail an environment (almost) devoid of stress, is what drives current research. Hence, this study aimed to examine the impact of a plant-based diet and simulated handling stress in fish farming on the microbiota associated with the intestinal mucosa in two genetic lines of rainbow trout. This well-defined resident microbiota is known to be essential to the host digestive system and is involved in several fundamental functions such as pathogen prevention, immune function, mucosal immunity, and nutrient metabolism^34^. Apart from that, it has been demonstrated that the intestinal gut microbiome differs between fish species or trophic levels^35,36^, inter-individual variation^37^, developmental stage or age, tissue of interest, including sub-compartments, health status, and dietary treatments, among others^38^. Considering those characteristics, mucus-associated bacteria are of major significance for the animal host, and thus, we analyzed those bacteria and, in a meta-analytical approach, compared them with lumen-associated bacteria from^33^ to gain a deeper understanding of the entire rainbow trout gut microbiota in relation to handling stress and feeding plant-based diets.

We did not observe any significant effect of the diet or the stress provided by chasing the fish on rainbow trout performance parameters in both trout genetic lines. The feed formulation in this trial was designed to meet all the nutritional requirements of rainbow trout and contained no limiting ingredients. The level of plant-based proteins was chosen so as not to affect growth performance, as the microbiome was to be studied under realistic feeding conditions with a plant-based diet. For a more detailed discussion of fish growth performance, please review^27^ and^33^.

It is known from several previous publications about rainbow trout^19,39–43^, Atlantic salmon^44–47^ and humans^48^, that the bacterial richness of the intestinal mucus layer is inherently low. Our results are in a similar size range and we further observed a significant association between decreased microbial richness in the gut mucus of fish fed with a diet composed of plant-based proteins in both trout genetic lines. In general, high microbial richness is assumed to have beneficial effects for the host since it comes with a higher metabolic capacity^49^. Previous studies reported that plant-derived proteins are able to increase the number of species observed in the gut. The decrease in microbial richness induced by the plant-based diet was thus an unexpected result (Fig. 1). On the other hand, the chasing, which was chosen to simulate handling stress in aquaculture like stocking, grading, sorting, and transporting, did not induce a significant change in alpha diversity. Nevertheless, the evenness expressed by the Shannon diversity index of the present species was unaffected by stress or diet in both trout genetic lines. As has been observed in several other studies, a general high level of variability between individuals within the respected treatments was observed.

Regardless of diet, stress, and trout line, pronounced differences in alpha diversity and microbial composition were observed between samples derived from intestinal contents^33^ and the intestinal mucus layer. Here, the intestinal content is marked by a four-fold higher richness (diff. in mean richness diet vs. mucus, p = 2.3e−21) in terms of ASVs compared to a general low microbial richness in the mucosal gut tissue. Those findings are in strong agreement with previous studies on the microbiota of mucus in rainbow trout^39,41,42,50^, Atlantic salmon^44,46,47^, or yellow catfish^40^. Otherwise, higher bacterial richness was observed in the mucosa of the middle intestine of common grass carp compared to the gut digesta^51^. Furthermore, higher richness values in the gut mucosa were also found in rainbow trout treated with *Pediococcus acidilactici* prebiotic compared to a control group^52^. However, low species richness in mucosal tissues is assumed to be characteristic and undermines accurately defined species composition. Beyond that, resident bacteria are supposedly maintained predominantly by host-related factors^13,53^, whereas otherwise transient digesta-associated communities are assumed to be more sensitive to environmental and dietary changes^41,54^. Our results do not confirm those statements, as the resident microbiota in the present study is predominantly impacted by dietary factors. An explanation for increased richness in intestinal contents might further be that the bacteria-rich chyme passes the intestine and is, according to that, a composition of bacteria derived from ingested microorganisms, bacteria derived from feed, and bacteria uncoupled from the mucosal community during the passage time of the digesta. The latter is represented by the fraction of 79 ASVs shared between the two compartments, while only a minor share is unique to the mucus compartment. The majority of the shared fraction has a consensus of 2–4 ASVs, implying that the similarity in gut mucosa and digesta lies in a minor count of bacterial genera and has high inter-individual variation.

Consistent with alpha diversity results, the composition of bacteria in the intestinal mucus microbiota of rainbow trout is primarily modified by the diet type in both trout genetic lines. However, we observed that these changes in the mucosal bacterial community due to the experimental diet are mediated by the handling stress, as significant differences between diets only occurred in stressed fish, as shown by pairwise comparisons of unweighted UniFrac distances. The modulation of the microbiota by stress involves complex interactions between the brain, the gut, and the microbiome, and there are several potential mechanisms. Stress in general activates the central nervous system, leading to the release of stress hormones such as cortisol^55^. Some bacteria are sensitive to cortisol, and alterations in hormone levels may create a selective environment for certain bacterial taxa. In Atlantic Salmon, for example, elevated fecal cortisol levels were associated with changes in the diversity and structure of the fecal microbiome, such as a decline in the lactic acid bacteria *Carnobacterium* sp. and an increase in OTUs within the classes Clostridia and Gammaproteobacteria^56^. The bacterial mucus community of our study was not generally affected by handling stress, but significant effects could be observed in combination with a change in the dietary protein source. A study in Atlantic cod juveniles demonstrated that tryptophan or phenylalanine supplementation significantly reduced plasma cortisol in cod juveniles after exposing them to acute stress^57^. Our experimental diets were designed to have an even amino acid profile meeting the nutrient requirements of rainbow trout (Supplements File S2 Table 6), however, the activity of digestive enzymes in the gastrointestinal tract can also be influenced by stress. For example, it was shown for Asian seabass in RAS that secretion and activity of digestive enzymes were significantly decreased with increasing stocking density (a chronic stressor)^58^. Stress-induced changes in protease activity, for example, may thus affect the efficiency of protein digestion and the availability of free amino acids for the organism or the intestinal microbiota. From our dataset alone, however, it is impossible to draw comprehensive conclusions on the physiological mechanisms behind these observations, and further investigations are needed. Nevertheless, a possible mechanistic connection between diet and stress is further supported by our previous findings, in which stress in trout genetic line A and an interaction of stress and diet in trout genetic line B were the key factors modulating the bacterial composition in gut contents^33^.

16S rRNA analysis identified *Firmicutes, Fusobacteriota, Proteobacteria, Desulfobacterota,* and *Spirochaetota* at the phylum level as the top five taxa in the intestinal gut mucosa of rainbow trout. The first three taxa are part of the core resident microbiota of rainbow trout^39,59,60^, whereas *Desulfobacterota* and *Spirochaetota* seem to be less observed in residential communities^61^. Furthermore, differences explained by the factor diet are within the abundance of bacteria from the genus *Mycoplasma,* which is more abundant in fish fed with plant-based proteins in trout line A and less pronounced in trout line B. As the most dominant genus in the phylum *Firmicutes*, *Mycoplasma* represented 52% (mean) of the bacteria in trout line A and 76% (mean) in trout line B. *Mycoplasma* has been identified as a key member of the healthy gut microbiota in the intestinal digesta^52,59,62–66^ and mucus^39,41,67^ of rainbow trout and Atlantic salmon^45,68–72^ using 16S rRNA amplicon sequencing. Huyben and colleagues^42^ revealed a similar high abundance of *Mycoplasma* in both the intestinal digesta and mucosa when reared under different temperatures, and a comparable high abundance was also found in the intestinal contents of two susceptible breeding lines of rainbow trout reared under different stocking densities^62^. Furthermore, a study investigating the adherent gut microbiota of rainbow trout fed with dietary insect meal from *Hermetia illucens* showed an increased abundance of *Mycoplasma* microbiota^73^, and the application of feed additive *P. acidilactici* MA18/5M also promoted an increase of *Mycoplasma* in the intestinal mucosa^52^. The highlighted recurrence of *Mycoplasma* implies a strong association between microbes and the gut microbiota of rainbow trout hosts under different conditions. In addition, its presence is associated with a positive health status, carotenoid utilization, and improved growth ^46,74^. Rasmussen and colleagues further proposed an improved metabolism for salmonids harboring *Mycoplasma*, explained by increased ammonia detoxification, better food utilization, and subsequent enhancement of growth. Interestingly, evidence was found that *Mycoplasma* promotes the degradation of long-chain polymers such as chitin, which explains its presence when an insect-meal diet is provided^75^. While *Mycoplasma* was highly abundant in both digesta and mucus, our results indicate a major presence of *Mycoplasma* in the gut mucosa, which underlines its highly commensal relationship to the host. Although gut mucosa harbors fewer different bacteria, bacteria from the genus *Mycoplasma* seem to colonize in high abundance, implying a potential beneficial function for salmonid hosts. Further investigations on the potential functions of *Mycoplasma*-related species beneficial for rainbow trout mucus microbiota need to be conducted by implementing meta-transcriptomics and/or metabolomics.

Notably, the increased abundance of *Mycoplasma* in fish fed diet V is accompanied by a decreased abundance of the luminous bacteria *Photobacterium* from the phylum *Proteobacteria* in trout line A. A similar ratio shift of *Firmicutes* and *Proteobacteria* in rainbow trout gut contents, though, was observed when fishmeal was entirely replaced by terrestrial proteins^76^. In trout line B, however, *Cetobacterium* seems to colonize the niche of *Photobacterium* when fed with a plant-based diet. It has been reported that *Photobacterium* is a common inhabitant of the alimentary canal of carnivorous fish^77^ and is rather promoted when fed with a fishmeal-rich diet, which is in strong agreement with the results of the present study. A study investigating the microbiome residing on feed in several aquaculture feeding experiments found *Photobacterium* to be a dominant genera, or at least remaining DNA, in the feed microbiome^78^. Although it is considered that the mucus microbiota is not significantly affected by transient DNA fragments, and observation of *Photobacterium* can be considered a true resident. However, some members, like *Photobacterium damselae,* are known to be pathogenic in aquacultured fish, including rainbow trout^79^. A similar increase of *Firmicutes* at the expense of *Proteobacteria* induced by plant ingredients was observed by Desai and colleagues^21^, and moreover, a similar ratio displacement of *Firmicutes:Proteobacteria* was observed in Atlantic salmon^68^ and chinook salmon^80^ associated with a fresh-to seawater transfer. Our results identified *Photobacterium* as the dominant representative of the phylum *Proteobacteria,* while the aforementioned studies revealed other bacterial species in this phylum. The recurring reciprocation between *Firmicutes* and *Proteobacteria,* here predominantly *Photobacterium*, might be explained by a growth inhibition of *Photobacteria* induced by *Firmicutes* bacteria (trout line A and references) or *Cetobacterium* as observed in trout line B when fed with a plant-based diet, which has been previously postulated by Zhao and colleagues^81^ in chinook salmon. Interestingly, the induction of *Photobacterium* by the primarily FM diet in trout line A in the present study was accompanied by proportionally higher expression levels of proinflammatory cytokines^27^. Upregulation of interleukin 1β plays an important role in the regulation of immune and inflammatory processes in the intestine (mucus) and has been observed during infections with potential pathogens in rainbow trout or gilthead sea bream^82,83^ or when exposed to stress. However, only some members of the genus *Photobacterium* are recognized as potential pathogens in multiple cultured fish species, and bacteria from the genus *Photobacterium* in our study could not be reliably assigned at the species level. We thus assume that the members of *Photobacterium* observed in the present study are either mutualistic candidates of the healthy carnivorous alimentary canal or that the quantity of *Photobacterium* is in a dimension of low danger of infection since no further indication of serious infection was observed.

As an exclusive representative of the phylum *Fusobacteriota*, we identified bacteria from the genus *Cetobacterium,* which is a common microbe in the intestine of fish like common carp^84^, bass^85^, and Nile tilapia^86^ and was identified as a dominant representative in carnivorous teleostei^53,85^. In rainbow trout, however, *Cetobacterium* prevalence was reported frequently^39,41,63,64,67^*,* but not to the extent seen in the present study. Here, *Cetobacterium* abundance is high in trout line A and less abundant in trout line B. An increase of *Cetobacterium* in fish fed a plant-based diet, independent of the applied stressor, as opposed to fish fed the FM diet in trout line B was observed, whereas in trout line A, a decrease associated with the plant-based diet was observed. Similar enrichment of *Cetobacterium* was observed in the intestinal contents of young zebrafish fed a diet high in soy protein concentrate^87^. In addition, Wang and colleagues postulate that *Cetobacterium* presence seems to be associated with improved glucose utilization mediated by acetate (and other short-chain fatty acids) accumulation. Another study, investigating five different freshwater fish, identified the vancomycin-resistant bacteria *Cetobacterium someare* as an indigenous colonizer of intestinal contents, which produces vitamin B12 to a great extent^88^ and represents a major source of vitamin B12 in fish metabolism since it remains an essential metabolite. Notably, *Cetobacterium* is a viable resource for beneficial metabolites such as butyrate, acetate, and propionate and suppresses the colonization of potential pathogens. Moreover, it seems to be associated with plant-based feeding, gut fermentation, vitamin B12 production, and the provision of enzyme activity^39,84^. We identified *Carnobacterium* as the only representative of lactic acid bacteria (LAB) in trout line A from treatment group 1-V. Bacteria from this genus are common in the gut microbiota of fish, and because of their beneficial role, they engage in gut health and protection against multiple fish pathogens in rainbow trout^89^. Additionally, they have been applied as probiotics in several aquaculture species^69,90,91^. LAB are characteristic for carbohydrate fermentation and are favored when feed stuff is rich in carbohydrates. However, soybean meal (5%) and wheat meal (11%) constitute the only carbohydrate sources in the provided plant-based diet, which is also supplemented in the FM diet (15%) and are most likely not considered the inducers of bacteria from this genus in treatment group 1-V.

This study explores the impact of a plant-based diet and simulated handling stress on the gut mucus microbiota in rainbow trout. Despite no significant effects on fish performance, the plant-based diet led to decreased microbial richness in the gut mucus. Stress did not influence alpha diversity but mediated changes in the mucosal bacterial community induced by the experimental diet. Notably, *Mycoplasma* dominated in the gut mucosa, showcasing its commensal relationship and potential health benefits for rainbow trout. *Photobacterium*, a Proteobacteria, decreased with the plant-based diet in trout genetic line A, while *Cetobacterium* increased in trout genetic line B. These changes suggest a reciprocal relationship between Firmicutes and Proteobacteria, potentially influencing the immune response. The study highlights the complex interplay between diet, stress, and mucosal microbiota and emphasizes the need for further research to unravel the underlying mechanisms. Our findings provide valuable insights into optimizing aquaculture practices by recognizing the nuanced relationships that shape fish gut health.

## Material and methods

### Ethics statement

All animal handling procedures were approved by the animal welfare officer of the Fraunhofer Research Institution for Individualized and Cell-based Medical Engineering (IMTE) (former: ‘Gesellschaft für Marine Aquakultur mbH’) and by the local authority of Schleswig–Holstein (MELUND, V 241–36,754/2018), according to relevant institutional and national guidelines for the care and use of laboratory animals (German animal welfare law; TierSchG and Regulation for the Protection of Animals Used for Experimental and Other Scientific Purposes; TierSchVersV as the national implementation of the Directive 2010/63/EU). The research also adhered to Aquaculture Research ethical guidelines and was conducted in compliance with the ARRIVE guidelines^92^.

### Experimental animals and feeding trials

All experimental procedures involving rainbow trout were operated at the IMTE facility in Büsum, Germany. The 59-day feeding trial was conducted in two identical recirculating aquaculture systems (RAS), each equipped with 20 tanks á 150 L, stocked with female rainbow trout from two different trout genetic lines (A and B). Trout genetic line A was acquired from Forellenzucht Trostadt (Forellenzucht Trostadt GbR, Tautenhahn, Germany), with its origin traced back to Troutlodge (Bonney Lake, USA). Trout genetic line B was purchased from Themar Fischzuchtanlage GmbH (Themar, Germany), and its origin can be traced to Frédéric Cachelou (Sarrance, France).

Twelve tanks of one RAS were stocked with 18 individuals from trout genetic line A (mean individual weight: 124.7 g ± 0.4), and twelve tanks of the second RAS were stocked with 16 individuals from breeding line B (mean individual weight: 147.2 g ± 0.7) to maintain a uniform stocking density of 15 kg/m3. Fish in both RAS experienced 15 h light exposition per day and were subjected to consistent environmental conditions (mean ± sd): water temperature (A: 15.17 °C ± 0.44, B: 15.42 °C ± 0.38), dissolved oxygen concentration (A: 9.67 ± 0.69 mg l^−1^, B: 9.47 ± 0.49 mg l^−1^), pH (A: 7.20 ± 0.40, B: 7.16 ± 0.23), salinity (A: 4.62 ppt ± 0.63, B: 4.56 ppt ± 0.61), ammonium (A: 0.43 mg l^−1^ ± 0.14, B: 0.50 mg l^−1^ ± 0.22), and nitrite (A: 0.96 ± 0.34 mg l^−1^, B: 1.07 ± 0.35 mg l^−1^).

Two experimental diets (F and V, feed formulation in Table 4), with equal nitrogen and energy content, were formulated and administered twice daily by hand (1.5% of tank-based group weight per day). Briefly, the fishmeal-based diet F contained 35% fish meal and the plant-based diet V contained 7% fish meal. In order to assess the impact of handling stress, half of the fish were chased twice daily for 60 s using an aquarium net 2 h after feeding (1), while the other half remained unexposed to this stress factor (0). Consequently, a total of eight experimental groups were established in triplicates (n = 3): A-0-V, A-1-V, A-0-F, A-1-F, B-0-V, B-1-V, B-0-F, B-1-F. A more detailed description of the experimental design can be found in^33^.

**Table 4:** Experimental diets F (FM-based) and V (plant-based) used in this study.

Ingredient	F [%]	V [%]
Fish meal LT70^a^	35.00	7.00
Fish protein concentrate	2.50	2.50
Soy protein concentrate^b^	6.00	20.50
Wheat gluten	6.00	15.20
Corn gluten	5.00	5.00
Soybean meal 48^c^	0.00	5.00
Wheat meal	15.00	11.40
Faba beans (low tannins)	6.00	6.00
Fish oil	13.92	14.64
Rapeseed oil	9.28	9.76
Vitamin and mineral premixtures INVIVO 1%	1.00	1.00
Vitamin C^d^	0.05	0.05
Vitamin E^e^	0.05	0.05
Antioxidant	0.20	0.20
Monocalcium phosphate	0.00	1.00
l-lysine	0.00	0.10
l-tryptophan	0.00	0.10
dl-methionine	0.00	0.50
Total	100.00	100.00
Nutritional composition (as fed basis)		
Water content	5.58	5.22
Ash content	7.74	4.85
Protein content	41.75	42.66
Fat content	26.50	26.48
Carbohydrate content	18.43	20.79
Gross energy [MJ kg^-1^]	23.54	23.92

The analytical components of the diet ingredients (% of original substance) have been provided by SPAROS Lda, Olhão, Portugal. Nutritional values and energy content was received at the GMA, Büsum.

^a^Peruvian fishmeal LT: 670 g kg^−1^ crude protein (CP), 90 g kg^−1^ crude fat (CF), EXALMAR, Peru.

^b^Soycomil PC: 630 g kg^−1^ CP, < 10 g kg^−1^ CF, ADM, The Netherlands.

^c^Solvent extracted dehulled soybean meal: 480 g kg^−1^ CP, 26 g kg^−1^ CF, SORGAL SA, Portugal.

^d^Vitamin C: > 35% sodium and calcium salts of ascorbyl-2-phosphate, BASF, Germany.

^e^Vitamin E: > 50% dl-alpha-tocopheryl acetate, BASF, Germany.

### Sampling procedures

Upon completion of the 59-day trial, the total intestines of 21 fish from each of the 8 treatments (7 fish per aquarium) was withdrawn to obtain gut content and mucus tissue. Therefore, fish were numbed by a blow to the head and subsequently euthanized by slitting the gill vein. The sample acquisition was conducted under sterile conditions to avoid contamination of the microbial communities by using sterilized scalpel blades and forceps. After carefully squeezing out the gut content (stored separately) and removal of remaining content material by rinsing the intestine with 10 ml sterile H_2_O (DECP treated; Carl Roth, Cat. no. T143.1) using sterile syringes, the mucus tissue was collected by carefully scraping the epithelial surfaces from the mucosal layer using a sterile plastic spatula. All samples were transferred to sterile Eppendorf tubes, placed on dry ice immediately and stored at − 80 °C until subsequent analysis.

### Bacterial DNA purification

Bacterial DNA obtained from the mucus layer of the intestines of rainbow trout from each treatment was purified using AllPrep^®^ PowerFecal^®^ DNA/RNA (Qiagen, USA, Cat. no. 80244) which enables simultaneous purification of microbial DNA and RNA from the same sample. As input, roughly 200 µL mucus was transferred into a microbial lysis tube and consequently lysed with a Precellys^®^ Evolution homogenizer (Bertin Corp., USA, Cat. no. P000062-PEVO0-A) for 45 s at 10.000 rpm. On a random basis, parallel to the biological samples, negative samples containing only lysis buffer were used as extraction controls. The rest of the purification steps were conducted according to the manufacturer’s instructions except for another 1-min incubation and elution step using the eluate generated with a volume of 30 µL buffer to increase the DNA yield. Until the samples were used for DNA sequencing, they were stored at − 80 °C.

### 16S rRNA library preparation and sequencing

For sequencing, the variable regions V3–V4 from 16S rRNA genes were amplified using the primer pair hV3F-hV4R^93^ in a dual barcoding approach. For amplification reactions and sequencing specifications, we refer to^33,94^. In addition to the biological samples, negative sequencing controls, positive controls (mock community, ZymoBIOMICS Microbial Standard), and extraction controls were sequenced on the Illumina MiSeq v3 2 × 300 bp (Illumina Inc., San Diego, CA, USA). Laboratory work, except for the DNA purification steps, was conducted at the Institute of Clinical Molecular Biology (IKMB, Kiel University).

### Bioinformatical processing

Following the demultiplexing procedure, read quality was examined using FastQC^95^ and MultiQC^96^. Processing of the sequencing reads was executed via the bioinformatics platform Quantitative Insights Into Microbial Ecology 2 (Qiime2 2021.2.0^97^). Read trimming of leftover primers and spacers was performed with the *qiime cutadapt trim-paired* command^98^. Chimera filtering, quality sorting, and merging of paired-end reads were conducted via the integrated DADA2^99^ pipeline with the command *qiime dada2 denoise-paired,* where the output is a feature-table with amplicon sequence variants (ASV). Accordingly, ASVs with a frequency of < 16 and an absolute abundance of < 2 per sample were filtered from the dataset. Taxonomic annotation was conducted by using a naïve Bayes classifier from the *qiime feature-classifier* plugin, previously trained with the SILVA database^100^ (release 138). ASVs classified as *Cyanobacteria* or mitochondrial DNA and ASVs with origins other than bacterial, were filtered out from further analysis. The taxonomic classification threshold was set at the phylum level. To conduct phylogenetic analysis of ASVs, a rooted tree was generated via the *qiime phylogeny align-to-tree-mafft-fasttree* command. The ASV abundance table, taxonomic information, and the rooted tree, together with a mapping file, were incorporated into the *phyloseq *^101^ package in R.

We also used 16S rRNA amplicon data generated from the intestinal gut content^33^, together with data obtained in this study, to identify differences in the microbiota between the two intestinal compartments (sample types). The data was evaluated only according to the sample type, diet, stress, and trout line parameters were ignored.

### Statistical evaluation and analysis

Alpha diversity, including the observed number of ASVs and Shannon diversity, was calculated using *phyloseq.* To determine statistical differences in alpha diversity, a Linear Mixed Effect (LME) model was defined^102,103^. The model included diet and stress and their interaction terms (twofold) as fixed factors, and tanks were incorporated as random factors. The residuals were assumed to be approximately normally distributed and heteroscedastic. Those conclusions were drawn based on graphical residual analysis. Based on this model, a Pseudo R^2^^104^ was calculated, and an analysis of variances (ANOVA) was conducted. Following the LME models, multiple contrasts were conducted to compare factor levels^105^.

A Welch two-sample t-test was used to compare means between the initial sampling of fish and all treatments combined, disregarding diet and stress within the respective trout line. Bacterial diversity between sample types of mucus and gut content was calculated as above. The Shapiro test was used to assess a normal distribution and the F-test to check for variance homogeneity. The non-parametric Wilcoxon rank sum test, used to compare bacterial diversity between sample types, and VENN diagrams were implemented with the phyloseq extension package MicEco^106^.

Non-metric multidimensional scaling plots were plotted by using weighted and unweighted UniFrac distances for diet and stress, respectively. To analyze data according to sample type (mucus and gut content) we use a Bray–Curtis dissimilarity matrix. All data used for Beta-Diversity analysis was Hellinger-transformed. Permutational analysis of variances (PERMANOVA) and analysis of similarities (ANOSIM) using the package vegan^107^ were used to test for statistical differences between groups. Multivariate homogeneity of groups dispersions (betadisper) was calculated using the same package. Following, pairwise comparisons were performed with the R package RVAideMemoire with no corrections for multiple testing, instead choosing only relevant groups, and p values were later manually adjusted using method *holm*. Boxplots were prepared using the ggplot package in R.

To determine which bacteria explain the differences in the experimental groups most probably, we conducted a linear discriminant analysis of effect size (LEfSe)^108^ with diet as a group factor based on beta diversity results. The analysis utilizes a nonparametric statistical test on individual taxa obtained from the given samples. Resulting statistical differences, indicated by an alpha value < 0.05 and an LDA log score of > 3, were then displayed in a bar graph. The same analysis was used with sample type as a group for mucus and gut content samples.

### Supplementary Information

Supplementary Figures.

Supplementary Tables.

## Data Availability

The datasets generated during and/or analyzed during the current study are available in the NCBI Sequence Read Archive (SRA) repository under the BioProject ID PRJNA797237 and the Study ID SUB13734035.
