# Supplementary material for: Effects of plant-based proteins and handling stress on intestinal mucus microbiota in rainbow trout
Source: Sci Rep. 2023 Dec 19;13:22563. doi: 10.1038/s41598-023-50071-x (PMC10728151; doi:10.1038/s41598-023-50071-x)
Supplement: Supplementary file 1 — Supplementary Figures. [file 41598_2023_50071_MOESM1_ESM.pdf]

Effects of plant-based proteins and handling stress on intestinal mucus microbiota in rainbow trout

Supplementary File S1

Marvin Suhr[1*], Finn-Thorbjörn Fichtner-Grabowski[1], Henrike Seibel[2], Corinna Bang[3], Andre Franke[3], Carsten Schulz[2,4], Stéphanie C Hornburg[1]

[1]Institute of Animal Nutrition and Physiology, Christian-Albrechts-University Kiel, Hermann-Rodewald-Straße 9, 24118 Kiel, Germany

[2]Fraunhofer Research Institution for Individualized and Cell-Based Medical Engineering (IMTE), Hafentörn 3, 25761 Büsum, Germany

[3]Institute of Clinical Molecular Biology, Christian-Albrechts-University Kiel, University Hospital Schleswig-Holstein, Rosalind-Franklin-Str. 12, 24105 Kiel, Germany

[4]Institute of Animal Breeding and Husbandry, Christian-Albrechts-University Kiel, Hermann-Rodewald-Straße 6, 24118 Kiel, Germany

Marvin Suhr: suhr@aninut.uni-kiel.de
Finn-Thorbjörn Fichtner-Grabowski: fichtner@aninut.uni-kiel.de
Henrike Seibel: henrike.seibel@imte.fraunhofer.de
Corinna Bang: c.bang@ikmb.uni-kiel.de
Andre Franke: a.franke@ikmb.uni-kiel.de
Carsten Schulz: c.schulz@tierzucht.uni-kiel.de
Stéphanie Céline Hornburg: hornburg@aninut.uni-kiel.de

* Corresponding author: Marvin Suhr (suhr@aninut.uni-kiel.de)

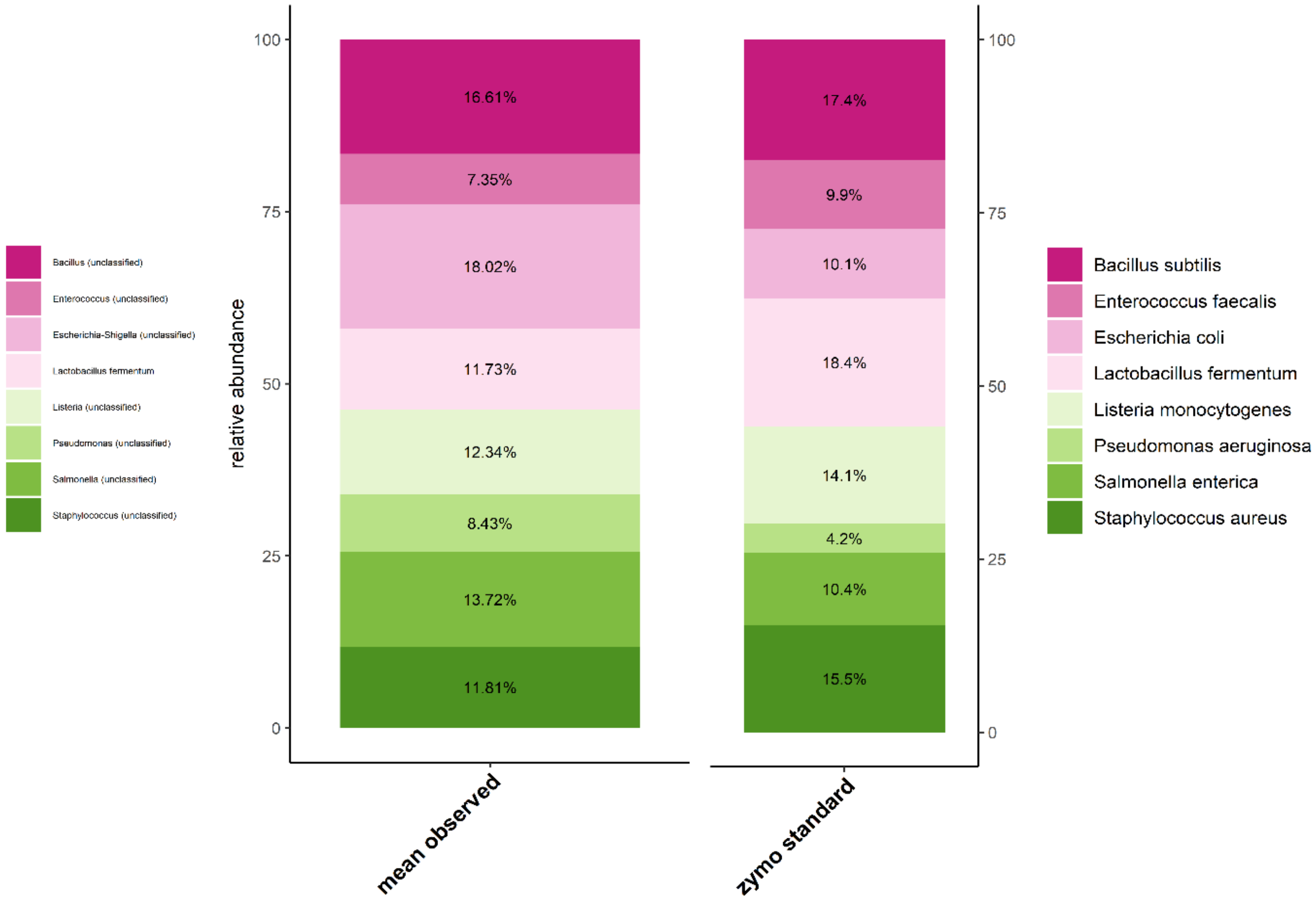

Figure S1 *Relative abundance of commercial community standard with mean observed (left bar) composition from sequencing and the expected composition from theoretical information of zymo standard (ZymoBIOMICS Microbial Community Standard, Cat. no. D6305, right bar).*

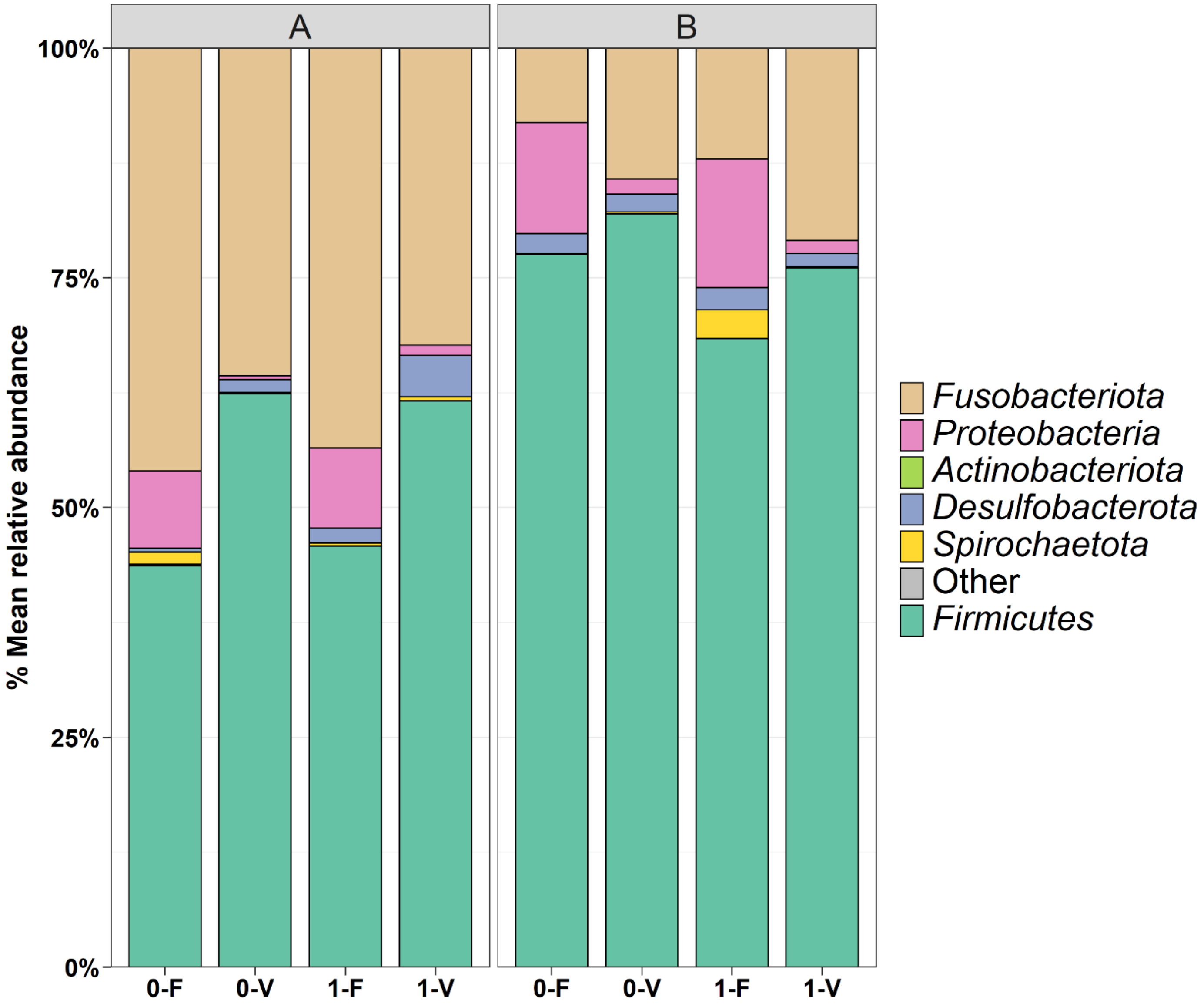

Figure S2 *Microbial composition of the intestinal mucus on phylum level. The four respective bars represent a diet-stress combination after the 59-day trial from both trout genetic lines, and the frequency of bacteria is indicated as the mean relative abundance. The data is based on the taxonomic rank phylum. Category 'Other' implies taxonomical clades with an overall abundance of < 0.15 %. The order of the bars is arranged by abundance, except for the most abundant genus, which is placed at the bottom for legibility. Each treatment incorporates individual fish data from the three (N=3) tanks. Unstressed group (0), stressed group (1), fishmeal diet (F), plant-based diet (V).*

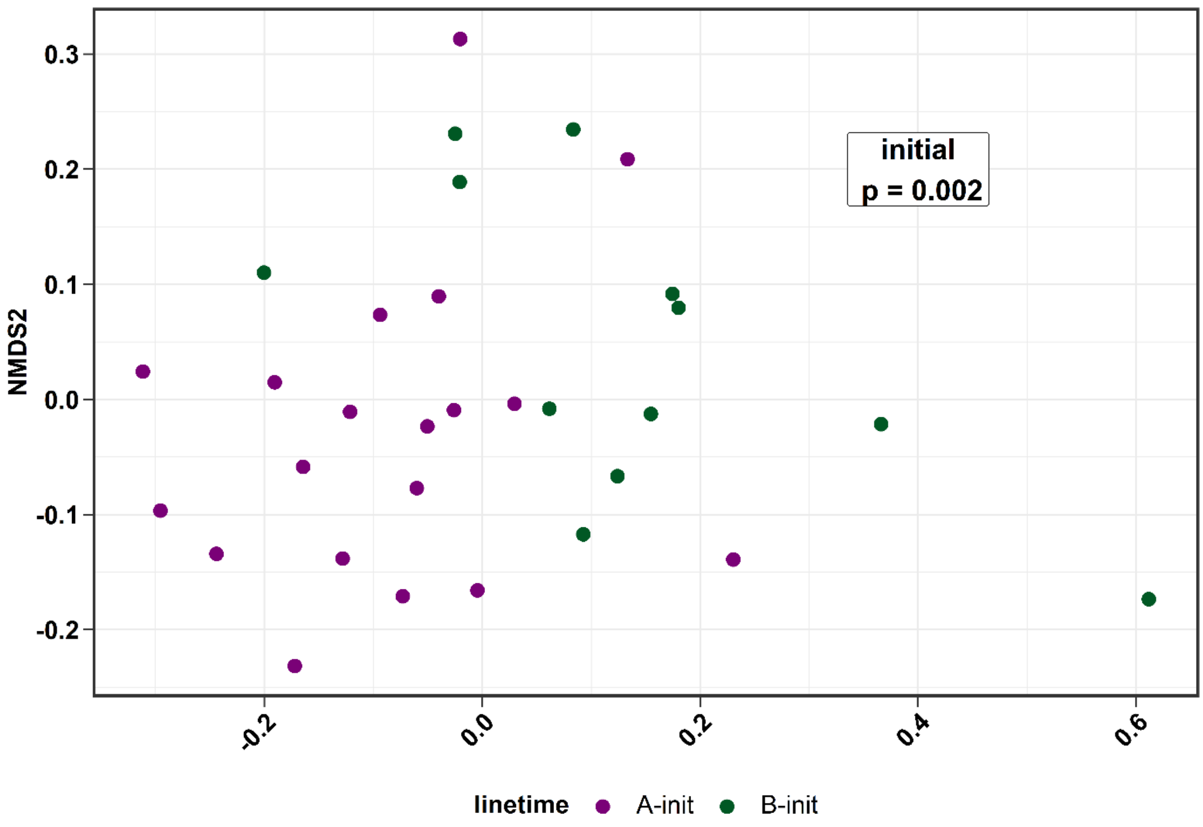

Figure S3 *Beta diversity of initial sampling of trout genetic line A and B using unweighted UniFrac distance with a significant p-value according to PERMANOVA.*

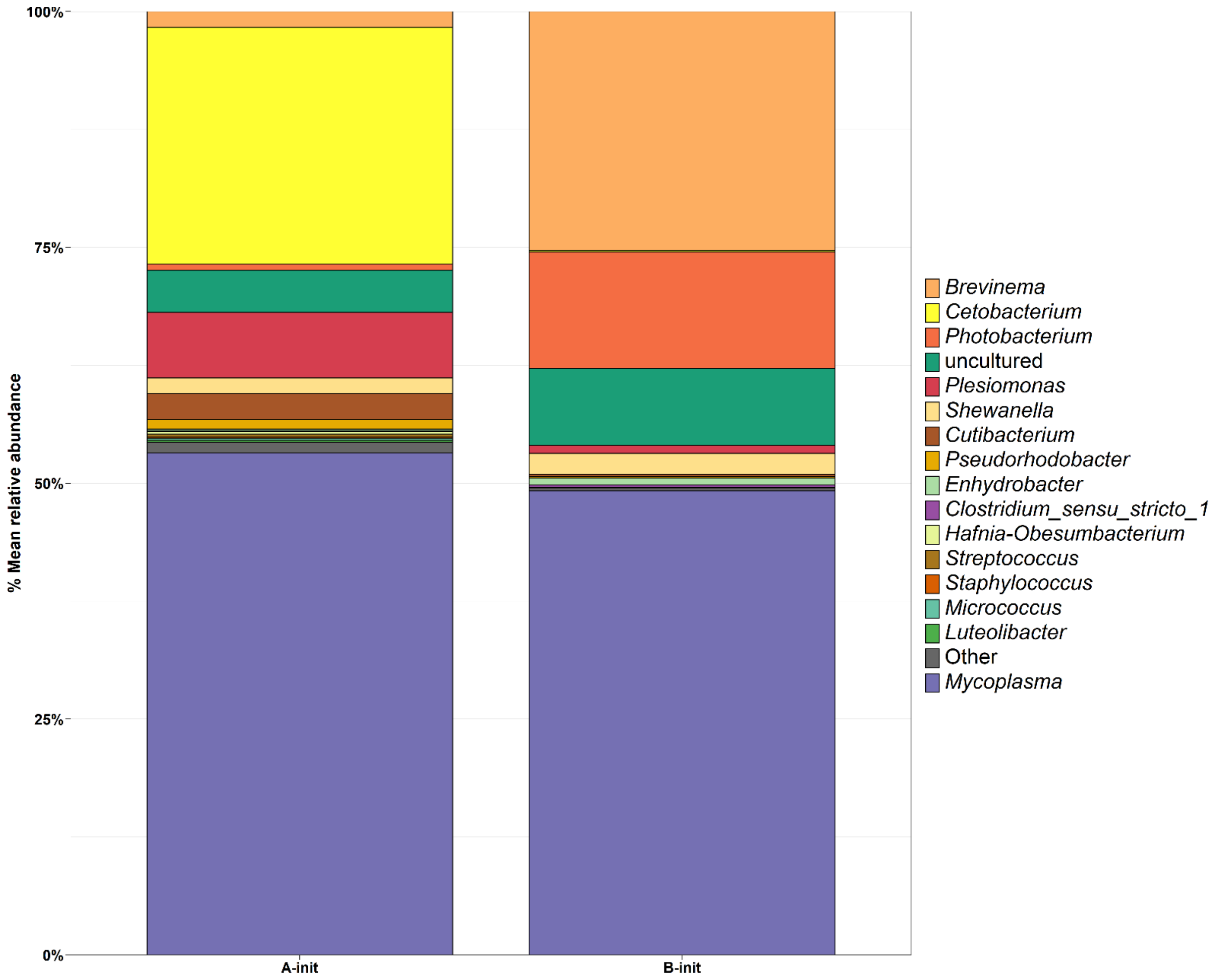

Figure S4 *Microbial composition of the intestinal mucus of initial sampling on genus level. The frequency of bacteria is indicated as the mean relative abundance. Category 'Other' implies taxonomical clades with an overall abundance of < 0.15 %. The order of the bars is arranged by abundance, except for the most abundant genus, which is placed at the bottom for legibility.*

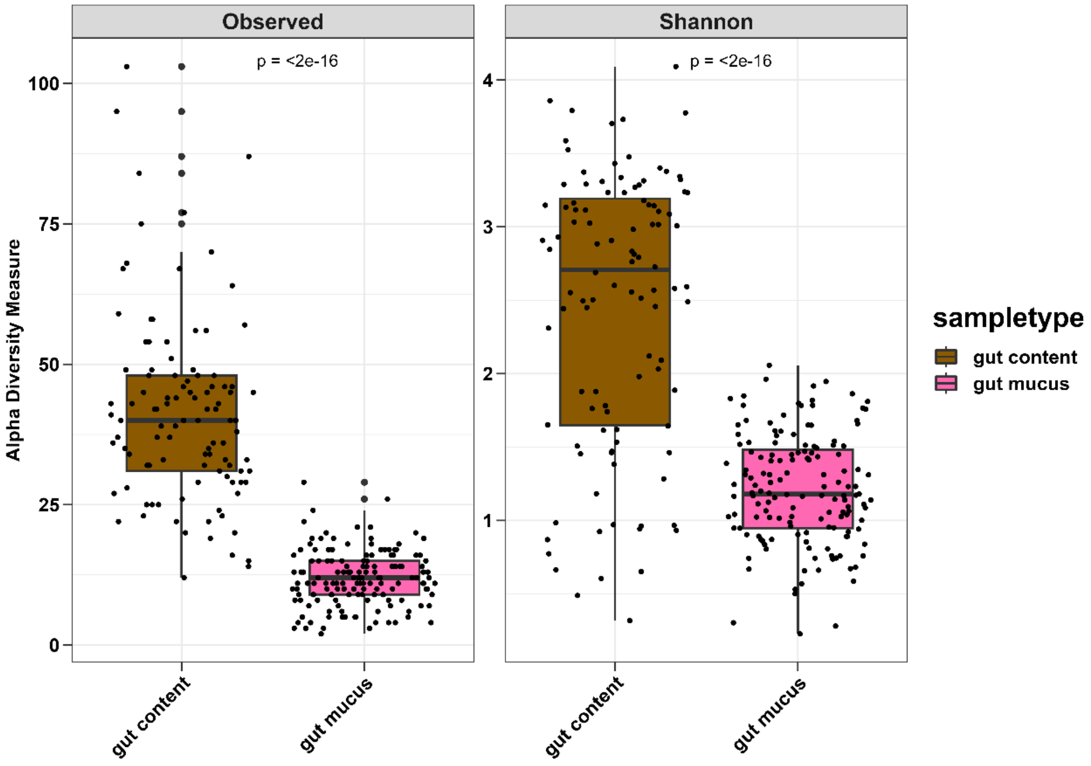

Figure S5 *Alpha diversity parameters Observed ASVs and Shannon diversity of the intestinal gut mucus and gut content indicating significant differences between both environments confirmed by non-parametric Wilcoxon test.*
